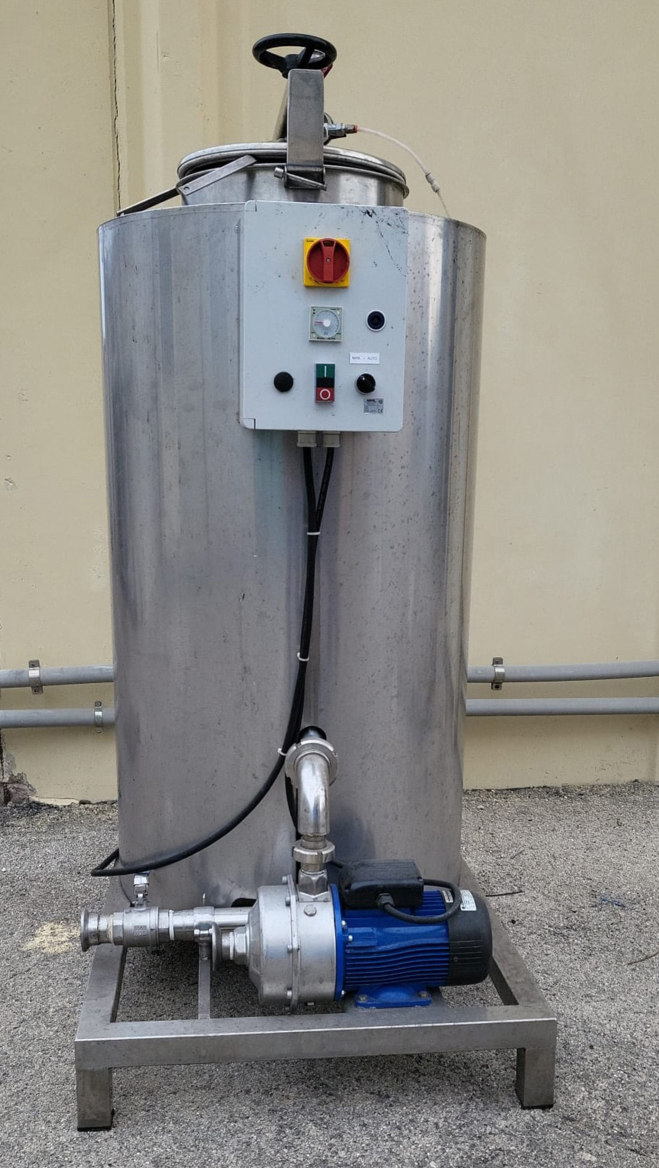# Low-pressure continuous dynamic extraction from oak chips combined with passive micro-oxygenation to tune red wine properties

**DOI:** 10.1016/j.heliyon.2024.e36100

**Published:** 2024-08-10

**Authors:** Vito Michele Paradiso, Gabriele Fioschi, Massimo Tripaldi, Luigi Sanarica, Chiara Pisarra, Mirella Noviello, Ilaria Prezioso, Giuseppe Gambacorta

**Affiliations:** a Department of Biological and Environmental Sciences and Technologies, University of Salento, S.P. 6, Lecce-Monteroni, I-73100, Lecce, Italy; b Centro Servizi Enologici, Sava, TA, Italy; c Enolife s.r.l., Montemesola, TA, Italy; d Department of Soil, Plant and Food Science (DISSPA), University of Bari Aldo Moro, Via Amendola, 165/a, I-70126, Bari, Italy

**Keywords:** Primitivo, Color stability, Medium chain fatty acids, Sorting, 5-Methyl-furfural, Furfural

## Abstract

Static infusion of oak chips in wine is a common practice during wine ageing, aimed at improving sensory properties and stability of wines. The wine/chips contact required to reach the desired effect can last several weeks or months. A low-pressure continuous dynamic (LPCD) extractor in which a closed-circle, low-pressure continuous flow of wine passes through an extraction cell filled with chips, was evaluated as a tool to tune red wine properties in few hours. The aim of this work was to evaluate the effect of the use of a LPCD extractor the effect on color, volatile compounds and sensory properties of a Primitivo wine, as well as to assess the combined effect of LPCD extractor, passive microxygenation through polyethylenetereftalate (PET) containers and exogenous tannins.

Their combined effect caused a significant increase of stabilized pigments was observed, without compromising the aroma profile.

LPCD extraction, passive micro-oxygenation through plastic materials and enological tannins can be considered as a low-cost, and potentially low-impact, integrated technological platform suitable to tune wine sensory properties and stability, when either traditional approaches (such as barrel aging) or other assisted extraction technologies are not applicable or preferred, even in small wineries.

## Highlights

• Low-pressure continuous dynamic (LPCD) extractor tuned wine properties in few hours.
• LPCD extractor improved sensory properties and color stability.
• Combined with passive micro-oxygenation in PET containers and enological tannin.
• These tools provide a low-cost integrated techonological platform for red wines.

## Graphical abstract

**Figure:**
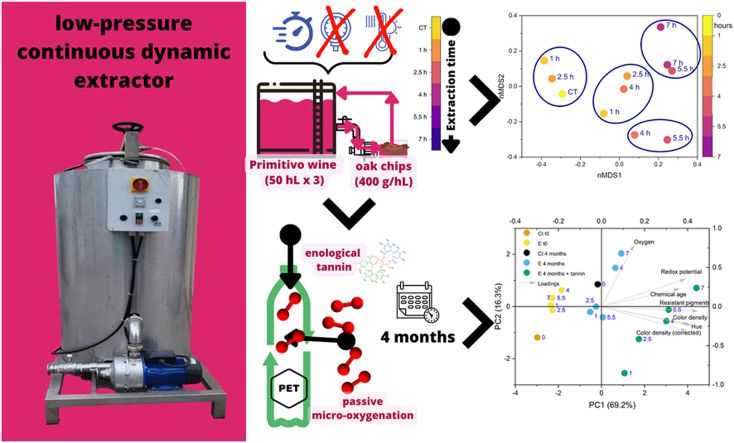

## 1 Introduction

“*Quidam […] gallas ustas immiscentes vino, permanens hoc faciunt*” (i.e.: “Some use to keep wine unaltered by mixing burnt galls with it”). So stated Burgundio of Pisa in his *Liber de vindemiis* in the XII century, translating on his turn the Greek book *Geoponica,* that dated back to the X century [1]. The use of baked or toasted oak pieces is, therefore, an ancient tradition as a mean to improve wine stability. Much more recently, the use of oak wood chips in winemaking has become an alternative practice to the use classic wood barrels, to exploit the interaction between wine and wood. Oak wood chips can be used in different phases of winemaking: during alcoholic fermentation, before malolactic fermentation or during wine aging. Delegated Regulation 2019/934 (EU) currently regulates their use in oenology [2]. The most used European species in oenology are *Quercus sessilis* and *Quercus robur* [3]. Oak chips are small pieces of wood with dimensions ranging from a minimum of 2 mm (called granulates, tabacs or wood rice) up to about 20 mm (called shavings, copeaux, or fragments), smaller size chips having more exchange surface area. Typical doses of use vary between 0.5 and 4 g L^−1^ for white winemaking and between 1 and 6 g L^−1^ for red winemaking and contact time typically ranges from a few weeks to a few months, depending on the size, and depending on the final product [4]. Oak wood chips are obtained from heartwood trees, whose main components are cellulose, hemicellulose and lignin. These components release several compounds, due to their hydrolysis or toasting; during toasting process guaiacol and derivatives are released from lignin that give smoky aromas [5]; furan compounds (furfural and 5-methylfurfural), released from cellulose and hemicellulose, confer almond aromas [[6], [7], [8], [9]]. Other important low molecular weight compounds are also released, such as acids, terpenes, volatile phenols, and lactones [[10], [11], [12]]. Hydrolyzable tannins are other important compounds extracted from wood. These are polymeric compounds derived from ellagic acid (ellagitannins) or gallic acid (gallotannins) and are transferred to the wine during aging, contributing to sensations of bitterness and astringency and behaving as antioxidants [[13], [14], [15]]. Moreover, oak tannins directly affect wine colour via reactions with anthocyanins forming complexes that are much more stable over time than free anthocyanins [14]. The aim of using oak wood chips is based mostly on avoiding high costs of oak wood barrels. Moreover, during the time barrels lose their potential release of aromatic components so reusing the same barrel for wine aging is limited. Finally, because of yeasts microbial activity on their surface, wood barrels could release undesirable compounds, such as volatile phenols, causing unpleasant odours [16].

Nevertheless, complete exploitation of oak chips oenological potential can be obtained coupling their use with wine micro-oxygenation, as an alternative to passive oxygen permeation through barrel wood. Micro-oxygenation, in fact, allows colour stabilization and mouthfeel improvement [[17], [18], [19], [20]]. Recently, other approaches for passive micro-oxygenation of wine, such as plastic materials, have been suggested [21].

Although oak wood chips contact time respect to aging in barrels can be 10 times shorter [22], new systems to further reduce time of process are being developed. This is essential to reduce economical costs and timely satisfy buyers’ demand. Assisted extraction can reduce contact time to few hours, due to accelerated extraction kinetics induced by physical means [23]. Hydrostatic pressure, mechanical waves (ultrasound) and electromagnetic waves (microwaves, pulsed electric fields) are the main physical means suggested for oak chips assisted extraction [[23], [24], [25], [26]]. Nevertheless, these approaches are not always suitable due to the high equipment cost and the side-effects that can occur in some cases [27,28]. Moreover, to the best of our knowledge, few data are available about the combination of assisted extraction and micro-oxygenation [29].

A low-cost, and potentially low-impact alternative approach to assisted extraction is low-pressure continuous dynamic (LPCD) extraction, based on a dynamic extractor in which a closed-circle, low-pressure continuous flow of wine passes through an extraction cell filled with chips. Therefore, the aim of this work was to evaluate the effect of the use of a low-pressure continuous dynamic extractor and of its interaction with passive microxygenation using polyethylenetereftalate (PET) containers and exogenous tannins on color, volatile compounds and sensory properties of a Primitivo wine.

## 2 Materials and methods

### 2.1 Materials and equipment

A wine from Primitivo grapes, aged one year in a steel tank, (2021 vintage, ethanol 15.1 % vol., total acidity 7.4 g L^−1^ tartaric acid, pH 3.5, total SO_2_ 86 mg L^−1^, free SO_2_ 19 mg L^−1^) was provided by La Popolare s.c.a. (Sava, Italy). Oak chips (INTENSE L, chips from *Quercus alba* L.) and oenological tannin (GRANROBUR TOSTATO obtained from *Quercus petraea* (Matt.) Loeb.) were provided by Enolife s.r.l. (Montemesola, Italy). The extractor used for low-pressure continuous dynamic (LPCD) extraction, model EXTW-200 (Fig. S1) was provided by Enolife s.r.l. (Montemesola, Italy). PET bottles (1 L) were provided by FA.MA.PET (Manduria, Italy).

### 2.2 Experimental plan

**Fig. 1:**
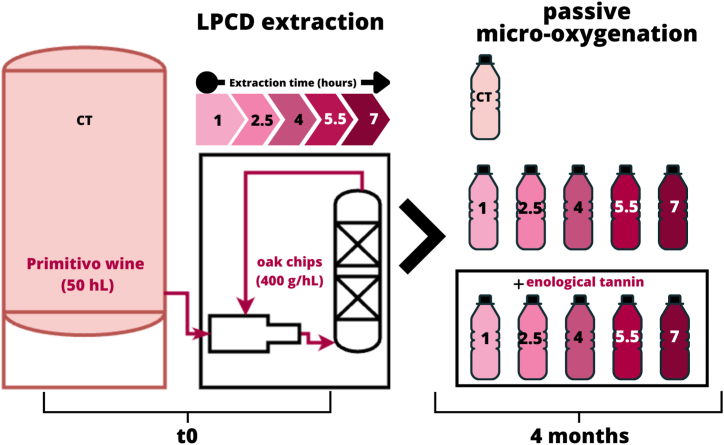
Experimental plan. CT, control wine; LPCD extraction, low pressure continuous dynamic extraction. The numeric labels indicate the duration of the extraction process (hours).

The experimental plan is reported in Fig. 1. Aliquots of 50 hL of wine were subjected to the treatment with the LPCD extractor, using 20 kg of chips (solid/liquid ratio of 4 g L^−1^). The treatment was carried out for 7 h, sampling the wine at predefined time intervals (1 h, 2.5 h, 4 h, 5.5 h, 7 h of treatment). The LPCD extractor operated in a pressure range of 20–30 kPa, with a nominal flow rate of 1.3 L s^−1^ at ambient temperature, with no appreciable increase of the wine temperature (14 ± 1 °C during the current experiment).

Control wine (CT t0) and treated wines (E t0 - 1 h, 2.5 h, 4 h, 5.5 h, 7 h) at each sampling interval were immediately analysed.

The remaining sampled wines were bottled 1-L PET containers to undergo passive micro-oxygenation. Each treated wine was bottled in two distinct bottles, one of which was also added with oenological tannin (0.1 g L^−1^). Control wine was also bottled in PET for comparisons. Bottles were then stored in dark at room temperature for 4 months. After storage, the control wine (CT 4 months), wines without tannin (E 4 months - 1 h, 2.5 h, 4 h, 5.5 h, 7 h) and wines with oenological tannin (E 4months + tannin - 1 h, 2.5 h, 4 h, 5.5 h, 7 h) were analysed.

The experimental plan was repeated in triplicate on three consecutive days on 50 hL aliquots of the same wine, directly in the cellar.

### 2.3 Chemical analyses

Oenological parameters were determined by infra-red analyser (WineScan, Foss, Hillerod, DK). Dissolved oxygen and redox potential were determined by a potentiometric portable analyser WTW Multi3630 IDS with probes FDO925 and IDR respectively (Thermo Fisher Scientific, Segrate, Italy). Measurements were repeated at intervals of 5 min for 30 min and the average value was considered. Color parameters were determined according to Mercurio et al. [30].

### 2.4 Analysis of oxygen permeation

Oxygen permeation in filled PET bottles was evaluated according to the following procedure. Three PET bottles were filled with a model wine (0.5 % w/v tartaric acid in 12 % v/v ethanol adjusted to pH 3.4 with 5 M NaOH, according to Mercurio et al. [30]). The model wine was degassed with a flow of nitrogen and bottles were closed and stored in dark at room temperature. The oxygen content in the model wine was measured according to 2.3 at filling and after 14 days. Possible oxygen consumption was monitored by acetaldehyde analysis, using a Hyperlab Smart enzymatic analyzer (Steroglass s.r.l., Perugia, Italy).

### 2.5 SPME-GC-MS analysis of volatile compounds

The volatile compounds were extracted by the solid-phase microextraction (SPME) technique, according to Ref. [31]. The samples were weighed (1 ± 0.05 ml) into 20 mL vials containing 0.2 g mL^−1^ of NaCl (to increase the ionic strength), closed with a silicone/PTFE septum and an aluminium cap. Semi-quantitation was performed adding internal standard (2-octanol). A mother solution obtained from the pure standard (Sigma Aldrich, Milan, Italy), was diluted to reach a final concentration of 8.2 mg L^−1^, then 10 μL of this final dilution was added to the sample. Samples were loaded into an autosampler Triplus RSH (ThermoFisher Scientific, Rodano, Italy). Before extraction, stabilization of the headspace in the vial was obtained by equilibration for 10 min at 50 °C. The extraction was carried out using a divinylbenzene/carboxen/polydimethylsiloxane (DVB/CAR/PDMS) 50/30 mm SPME fiber assembly (Supelco, Bellefonte, PA, USA) at 50 °C for 30 min. The fiber was desorbed at 200 °C for 2 min in the injection port of the gas chromatograph, operating in split-less mode. The GC-MS analyses were performed using a Trace1300 gas chromatograph equipped with a mass spectrometer ISQ Series 3.2 SP1. The compounds were separated in a Thermo capillary column VF-WAX MS (60 m, 0.25 mm, 0.25 mm), under the following conditions: injection port temperature, 200 °C; oven temperatures, 40 °C for 0.5 min then 3 °C min^−1^ to 210 °C with a final isothermal for 2 min. Mass detector was set at the following conditions: detector voltage, 1700 V; source temperature, 250 °C; ionization energy, 70 eV; scan range, 33–150 amu. Tentative identification of the peaks was done by means of Xcalibur v2.0 software, in particular, Qual Browser, by matching their spectra with the reference mass spectra of NIST library. Semi-quantitation of the compounds was done by the internal standard method, and the amounts were expressed as mg of 2-octanol equivalents L^−1^.

### 2.6 Sensory analysis

A panel composed of 8 judges (6 males, 2 females; age 23–60), winemakers and professionals, participated at wine evaluation sessions. All judges were experienced in wine tasting and were familiar with Apulian cultivars. One training session was carried out to familiarize with the sensory methodology. No human ethics committee or formal documentation process is available. All participants were volunteers and before participating in the study they signed an informed consent form defining the type of research and voluntary participation. All data were collected anonymously. Wines were presented in random order at serving temperature (17 ± 2 °C) in ISO-standard glasses [32] coded with 3-digit random numbers.

#### 2.6.1 Sensory analysis of wines at t0

Samples from LPCD extraction were first characterized at time 0 by a free sorting task [33]. The control wine and two samples from independent trials per each extraction time were presented to panelists. Assessors were asked to arbitrarily group samples according to their similarity for aroma and taste. The data obtained were analysed as described in the *Statistical analysis* section (2.7).

One sample per each time of extraction, randomly picked among the independent replicates, was then submitted to quantitative descriptive analysis (QDA). Panelists evaluated wines on a 10-cm unstructured scale for the following descriptors: fruity, floral, vegetal, wood, spicy, caramel/toasted, aroma intensity, aroma quality, body, sweetness, acidity, bitterness, astringency.

#### 2.6.2 Sensory analysis of wines after micro-oxygenation

Based on the results of the free sorting, only representative times of extraction were considered for QDA after micro-oxygenation, considering the high number of wines, given the further variable of the oenological tannin. Therefore, wines from 4 to 7 h of LPCD extraction, both without and with tannin were analysed and compared with the control wine after micro-oxygenation. One sample, randomly picked among the independent replicates was analysed as in paragraph 2.5.1.

### 2.7 Statistical analysis

The data of the chemical parameters (section 2.3) were analysed with two-way Analysis of Variance (ANOVA) with Tukey's HSD post-hoc test for multiple comparisons to evaluate the effect of time of treatment and of storage (passive micro-oxygenation and tannin addition). Moreover, correlation analysis was carried out at t0 to evaluate correlations among duration of treatment and chemical parameters. Finally, principal components analysis (PCA) was carried out to highlight changes on the chemical pattern.

PCA was also applied to the volatile profile (section 2.4) to highlight the changes on the pattern of volatiles.

The data deriving from the free sorting task (section 2.5.1) were used to obtain contingency tables reporting the frequency of the appearance of each pair of samples in the same group, and non-metric multidimensional scaling was the performed [34] to project samples similarity/dissimilarity on a two dimensional plane. Hierarchical clustering analysis was further applied to highlight groups of similarity [35].

The data of QDA (sections 2.5.1 and 2.5.2) were analysed by PCA. Moreover, correlation analysis was carried out at t0 to evaluate correlations among duration of treatment and sensory descriptors.

Finally, partial least squares regression (PLSR) analysis was applied to the datasets of QDA and volatile compounds to explore relations existing between the volatile profile and aroma descriptors.

All statistical analyses were carried out with Origin Pro 2023 (OriginLab, Northampton, MA, USA).

## 3 Results and discussion

### 3.1 Redox conditions and color parameters

**Fig. 2:**
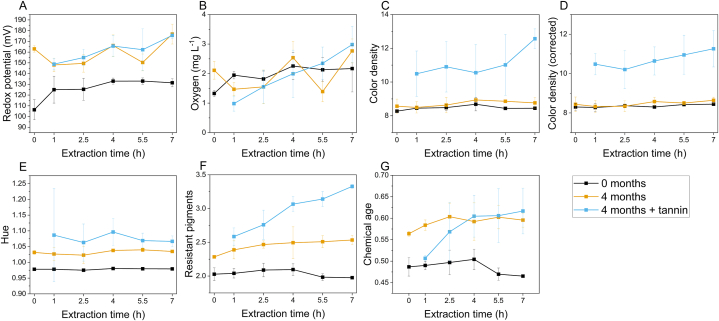
Indices of redox conditions and color in the Primitivo wine, as a function of the duration of dynamic extraction, after treatment (t0) and after four months of passive micro-oxygenation, both without (4 months) and with added tannins (4 months + tannin). A, redox potential as a function of extraction time; B, dissolved oxygen as a function of extraction time; C, color density as a function of extraction time, D, color density (corrected for SO_2_) as a function of extraction time; E, hue as a function of extraction time; F, SO_2_ resistant pigments as a function of extraction time; G, color chemical age as a function of extraction time.

Fig. 2 reports the values of the indices of redox conditions and color in the Primitivo wine as a function of the duration of LPCD extraction, after treatment and after four months of passive micro-oxygenation, both with and without added tannins. Results of two-way ANOVA are reported in Supplementary Table S1.

**Fig. 3:**
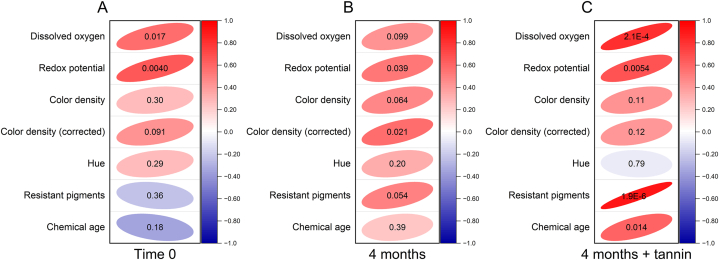
Analysis of Pearson's correlation between the duration of the dynamic extraction process and the indices of redox conditions and color parameters of wines after treatment (A, Time 0) and after 4 months of passive micro-oxygenation, without (B, 4 months) and with added tannins (C, 4 months + tannin).

Fig. 3, instead, reports the results of the analysis of Pearson's correlation between the duration of the LPCD extraction process and the indices of redox conditions and color parameters of wines after treatment and after 4 months of passive micro-oxygenation, with and without added tannins.

Both redox potential (Fig. 2A) and dissolved oxygen content (Fig. 2B) showed increases after LPCD extraction ranging respectively 19–27 mV and 0.6–0.9 mg L^−1^ compared to the control wine. They were positively correlated in treated wine with the duration of treatment with the dynamic extractor (*p* < 0.05 and *p* < 0.01, respectively, Fig. 3A). The increase of oxygen is congruent with data reported in literature for other aging/fining operations [36]. The relatively low redox potential values observed can be considered appropriate for a red wine aged for one year in steel tanks [37,38].

The scenario after 4 months of micro-oxygenation resulted quite complex. The oxygen transmission rate of PET bottles reported in literature is variable. Giovanelli and Brenna reported 0.0210 mL bottle^−1^ day^−1^, corresponding to about 0.028 mg bottle^−1^ day^−1^ (density 1.317 kg m^3^ at 1 atm and 23 °C) and to about 0.097 mg L^−1^ day^−1^ [39]. Mentana et al. instead, measured an oxygen transmission rate in PET bottles used for Apulian red wines of 0.089 cm^3^ bottle^−1^ day^−1^, corresponding to about 0.117 mg L^−1^ day^−1^ [38]. Toussaint et al. estimated oxygen ingress in PET bottles containing rosé wine between 25 and 30 mg bottle^−1^ year^−1^, corresponding to 33.33–40 mg L^−1^ year^−1^ [40]. The oxygen permeation observed in our experiment with a model wine (Supplementary Table S2), with negligible oxygen consumption (confirmed by the analysis of acetaldehyde) was higher (0.23 ± 0.08 mg L^−1^ day^−1^). Considering that a suggested amount of oxygen supplied by micro-oxygenation ranges from 40 mg L^−1^ year^−1^ [17] to 1.3–6.6 mg L^−1^ month^−1^ during 1–4 months [18], storage in PET bottles can be considered to supply similar or slightly higher amounts of oxygen as micro-oxygenation. However, as regards dissolved oxygen, no significant change was observed in our experimental wines after 4 months (Fig. 2B), probably due to the complex balance of dissolved oxygen consumption, headspace oxygen solubilization and atmospheric oxygen permeation; moreover these phenomena occur with varying intensity during storage in PET containers [40]. Nevertheless, also for dissolved oxygen a strong positive correlation (*p* = 0.00021) was observed with the duration of dynamic extraction process when oenological tannins were added (Fig. 3C).

The redox potential in the wines was significantly higher after 4 months of storage in PET bottles (Fig. 2A). The average increase was about 35 mV. As recently reported by Danilewicz et al. [41], the redox potential measured on platinum electrodes is largely due to oxygen reduction and ethanol oxidation, though other redox couples can contribute to the measured potential at low oxygen levels. Therefore, the increase can be explained with both the solubilization of oxygen from headspace and permeation through PET [40], but the formation of high-redox potential quinones from catechol oxidation cannot be excluded [42]. Positive correlations (*p* < 0.05 and < 0.01 without or with tannin respectively, Fig. 3B and C) were observed with the duration of the treatment, suggesting the involvement of redox-active compounds from oak chips in the formation of quinones. No significant difference was pointed out by two-way ANOVA between wines with and without oenological tannin.

As regards color, Fig. 2 reports the values of color density, color density corrected for decoloration due to SO_2_, and hue (panels C, D, E respectively). Slightly positive correlations were observed for color density and corrected color density with the duration of LPCD process (Fig. 2C and D), though the only significant correlation was found for corrected color density after 4 months without tannin (Fig. 3). This suggests possible rapid effects on color stabilization and intensification with the dynamic extraction. However, the effect of longer extraction processes was reduced during storage. By far higher was the impact of the addition of tannins, that determined color density values increase on average by 29 % (Fig. 2C). Though ellagitannins are known to contribute to color stabilization, due to their redox activity [43], the effect observed in this study was higher than reported in previous literature and could be due to a synergic effect of chip treatment, oxygen solubilization and exogenous tannin [[44], [45], [46]].

The hue of wines (Fig. 2E) significantly increased after 4 months, particularly in presence of exogenous tannin, with values significantly higher than in wines without addition. No correlation was observed with the duration of LPCD extraction (Fig. 3). This change of hue, expected during wine ageing, can be related to the formation of polymeric pigments and pyranoanthocyanins, enhanced by the addition of tannins and presumably by the availability of small amounts of oxygen [47]. Moreover, the addition of oenological tannin could have led to the formation of anthocyanin-ellagitannin complexes, characterized by a bathochromic shift of the visible spectrum [48].

This is confirmed by the values of resistant pigments (Fig. 2F) and chemical age of color (Fig. 2G) observed in the wines. These indices are obtained from the absorbance at 520 nm of the wine diluted in a buffer containing sodium metabisulphite in excess [30,49], and have been related to the content of polymerized pigments resistant to the bleaching effect of sulfur dioxide. Both indices increased during storage and reached significantly higher values in wines with added tannins. Moreover, in this case significant correlations were observed with the duration of extraction process (Fig. 3C). These results point out the color stabilization phenomena occurred in bottles, with an interaction of added tannins with phenolic compounds extracted from oak chips. Picariello et al. [46], analogously reported the effectiveness of ellagitannins in increasing polymeric pigments in red wines under oxidative conditions.

**Fig. 4:**
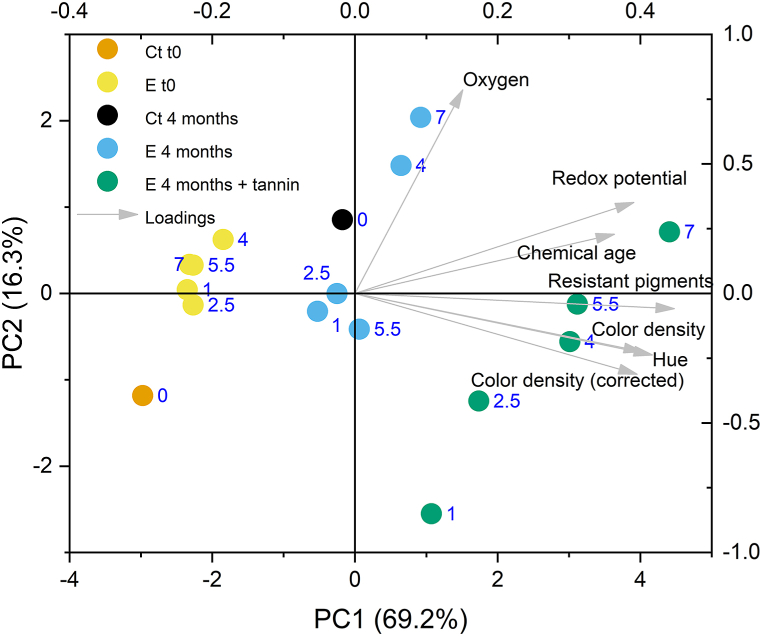
Principal components analysis of the data of redox indices and color parameters of wines. Ct t0, control wine at time 0; Ct 4 months, control wine after 4 months micro-oxygenation; E t0 wines after dynamic extraction; E 4 months, wines after dynamic extraction and 4 months of passive micro-oxygenation; E 4 months + tannin, wines after dynamic extraction and 4 months of passive micro-oxygenation with added tannins. The numeric labels indicate the duration of the extraction process (hours).

A clear overview of these data is provided by principal components analysis (Fig. 4). The principal component (PC) 1 explained almost the 70 % of the data variability and was mainly correlated with all the indices of aging and color stabilization.

The PC2 accounted for the 16.3 % of variability, showed a strong positive correlation with dissolved oxygen, and could be also related and to a shift from color density and stability to oxidative phenomena (i.e.: redox potential and chemical age). Along PC1 the samples were grouped according to PET bottle aging and use of tannin. Compared to micro-oxygenation alone in PET bottles, micro-oxygenation coupled with added tannins provided an acceleration of the aging process. Moreover, scores of PC1 slightly increased as LPCD extraction time increased. Wines submitted to increasing LPCD extraction time also scattered along the PC2. Just after extraction, treated wines were grouped along PC2 separately from the control wine. Greater dispersion of the PC2 scores was observed in wines after 4 months of storage in PET bottles, without apparent relation with the LPCD extraction time. On the contrary, the PC2 scores of wines with added tannins were highly correlated (*p* = 0.002) with the extraction time. PCA, therefore, highlights the modulatory effect of the tested tannin on the oxidative phenomena connected to color stabilization.

### 3.2 Volatile profile

The SPME-GC/MS analysis of volatile compounds allowed to focus on 36 compounds, including four aldehydes, six alcohols, seven ethyl esters, five acetate esters, two other esters, two terpene compounds, four acids, four furan compounds, one lactone and one sulfur compound (Supplementary Table S3).

**Fig. 5:**
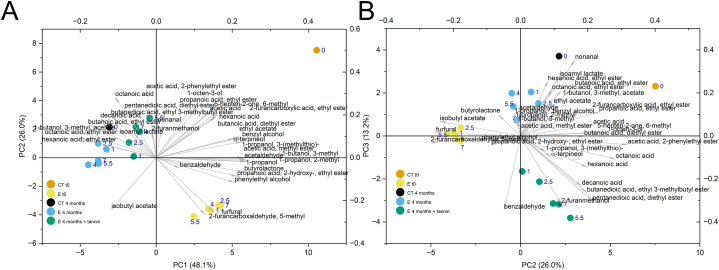
Principal components analysis of the data of volatile compounds of wines (A, biplot of PC1 and PC2; B, biplot of PC2 and PC3). Ct t0, control wine at time 0; Ct 4 months, control wine after 4 months micro-oxygenation; E t0 wines after dynamic extraction; E 4 months, wines after dynamic extraction and 4 months of passive micro-oxygenation; E 4 months + tannin, wines after dynamic extraction and 4 months of passive micro-oxygenation with added tannins. The numeric labels indicate the duration of the extraction process (hours).

An overview of the effect of LPCD extraction, passive micro-oxygenation and tannin on the volatile profile was obtained by multivariate analysis. The PCA provided the results reported in Fig. 5. The first three principal components accounted for 85 % of data variability. The score plot of the first two principal components mainly highlighted the effect of LPCD extraction treatment and aging (Fig. 5A). The untreated wine (CT t0) scored on the top right quadrant, related to several volatile compounds typical of young red wines mainly including esters and higher alcohols. The LPCD extraction from chips was strongly related to the content of furfural and 5-methyl-furfural, as expected [50]: the treated wines were accordingly clustered in the bottom-right quadrant. Furfural and 5-methyl-furfural are reported as characterizing volatiles extracted from medium-to-high toasted chips [51]. Passive micro-oxygenation determined a shift of the volatile pattern, clustering all the wines in the top-left quadrant of the bi-plot. The volatile compounds characterizing these wines included several ethyl esters of short- and above all medium-chain fatty acids. Literature reports that the equilibria of hydrolysis and synthesis of ethyl esters can determine decreases as well as increases of these compounds during wine ageing [[52], [53], [54], [55]]. The addition of oenological tannin to wines reduced the extent of this shift in the pattern of volatiles. Moreover, the PC3 (Fig. 5B) highlights an interesting effect of chips and oenological tannin. In fact, the PC3 axis is positively correlated with ethyl esters and negatively correlated with medium chain fatty acids. The addition of tannin determined a shift towards acids rather than their esters.

**Fig. 6:**
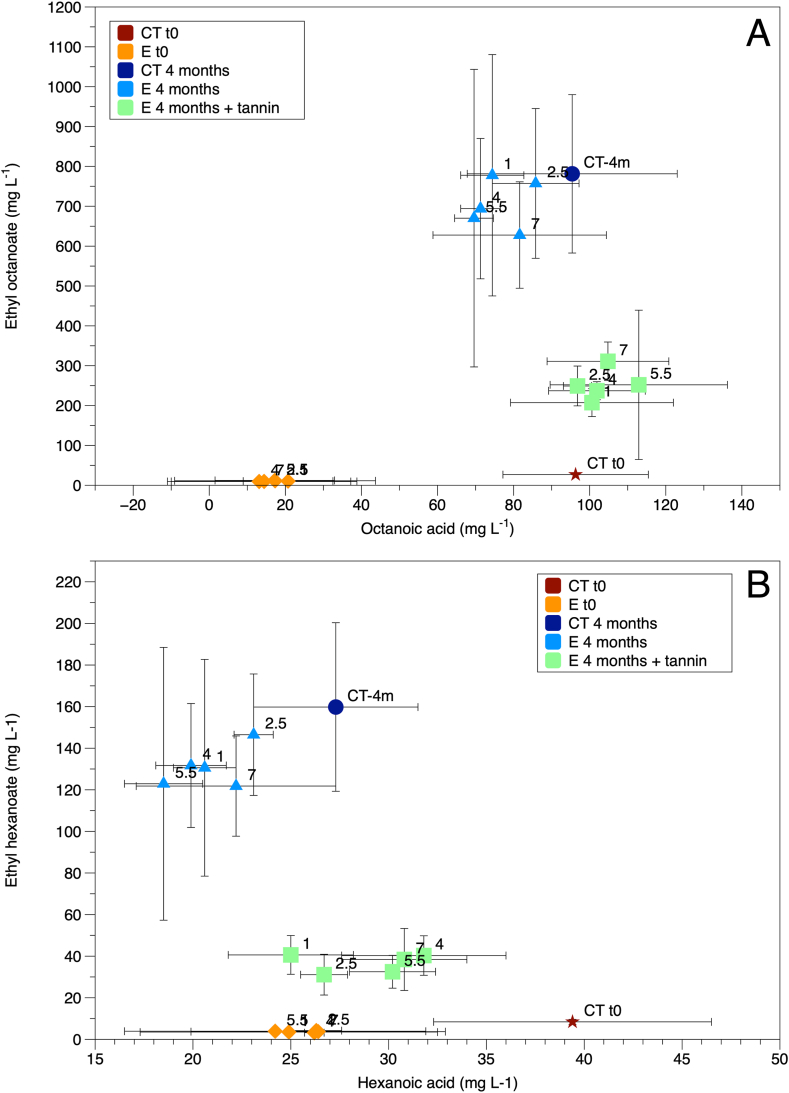
Levels of C8 (A) and C6 (B) organic acids and their ethyl esters in the experimental wines. Ct t0, control wine at time 0; Ct 4 months, control wine after 4 months micro-oxygenation; E t0 wines after dynamic extraction; E 4 months, wines after dynamic extraction and 4 months of passive micro-oxygenation; E 4 months + tannin, wines after dynamic extraction with added oenological tannin. The numeric labels indicate the duration of the extraction process (hours).

Fig. 6 reports in the panels A and B respectively the levels of C8 and C6 acids and their ethyl esters. Compared to control wine (CT t0), the LPCD extraction from oak chips (E t0) determined a decrease of the acids, irrespective of the treatment time, probably due to sorption phenomena onto chips, whilst esters did not substantially change. After passive micro-oxygenation in PET bottles, increases of both esters were observed, while the acids did not show an opposite trend as could be expected when considering only esterification/hydrolysis equilibria. Moreover, the effect of oenological tannins in reducing the increase of esters suggests that possible oxidation pathways could have influenced the acid/ester equilibria. In particular, we hypothesize that the hydrolysis/esterification equilibria involving the ethyl esters of medium chain fatty acids could be affected by other phenomena, i.e. ex-novo formation of acids via chemical oxidation of long-chain fatty acids contained in oak wood and released in wine by chips [11,[56], [57], [58]].

Oenological tannin could have determined a delay in formation and, therefore, in the subsequent esterification of medium chain fatty acids. Further investigation is required to confirm this hypothesis.

### 3.3 Sensory analysis

**Fig. 7:**
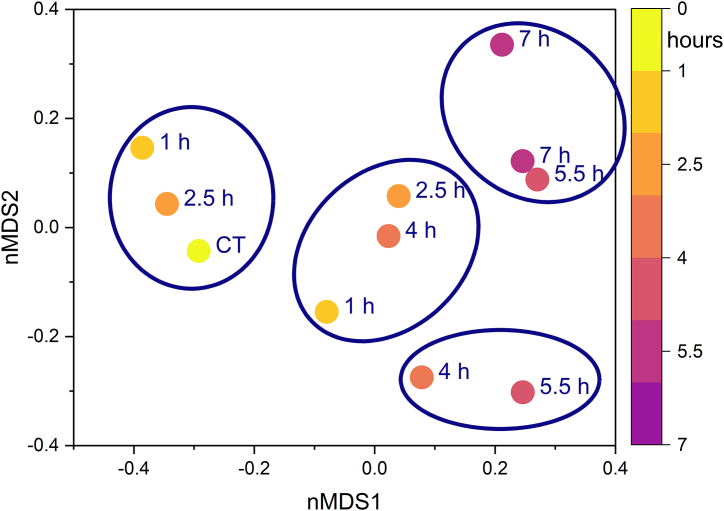
Non-parametric multidimensional scaling applied to the contingency tables deriving from the sorting analysis carried out by the sensory panel on the wines after dynamic extraction. Ellipses include clusters obtained by hierarchical clustering applied to plot coordinates. CT, control wine. Numeric labels indicate the duration of the extraction process.

Free sorting analysis was carried out by the sensory panel on the control wine and wines from two independent trials of LPCD extraction. The resulting contingency tables, reporting the frequencies of co-occurrence in the same group for each pair of the analysed wines, were submitted to non-parametric multidimensional scaling (nMDS). The plot of the first two dimensions (Fig. 7) graphically represents similarities and dissimilarities among the tested wines as distances on the plot. Hierarchical clustering analysis was subsequently applied to samples' scores, in order to obtain clusters of similar wines according to panelists’ perception [59].

The clustering of wines highlights that a clear discrimination could not be observed among LPCD treatments differing for less than 3 h of duration. The sensory impact of time of LPCD treatment can be therefore considered as a continuous variable, modulable by varying the duration of LPCD extraction.

**Fig. 8:**
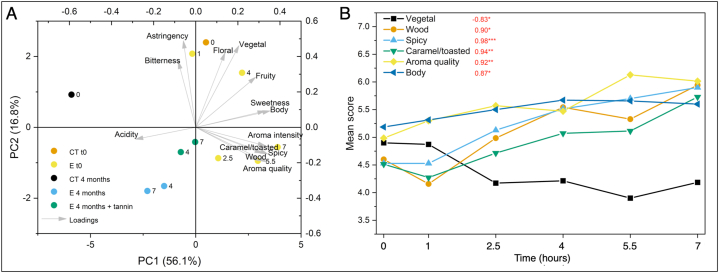
Principal components analysis of the data of QDA sensory analysis of wines (A) and plot of descriptors of wines at time 0 against extraction time (only descriptors with significant correlation are plotted, B). CT t0, control wine at time 0; CT 4 months, control wine after 4 months micro-oxygenation; E t0 wines after dynamic extraction; E 4 months, wines after dynamic extraction and 4 months of passive micro-oxygenation; E 4 months + tannin, wines after dynamic extraction with added oenological tannin. The numeric labels indicate the duration of the extraction process (hours). Perason's r value is reported in red figures.

Fig. 8A reports the results of the PCA applied to the mean scores obtained from the QDA carried out on the wines after LPCD extraction and on the wines selected for QDA after micro-oxygenation; The first two principal components from PCA accounted for 72.9 % of data variability (Fig. 8A). Wines submitted to low-pressure dynamic extraction scattered along the PC2 (16.8 % variability). Fig. 8B reports the plot of the descriptors of wines at time 0, against LPCD extraction time (only descriptors with significant correlation are plotted). Increasing extraction time could be related with sensory descriptors, as pointed out by the value of Pearson's *r*. In particular, increasing LPCD extraction time determined decreasing perception of vegetal notes, as well as increasing aroma quality together with wood, caramel/toasted and spicy notes. Also, higher perception of body could be related to high extraction times. The effect of contact with oak chips on similar descriptors is well known [56,60,61]. On the other hand, a significant decrease of herbaceous notes was already reported by Cano-López et al. [62]. The increasing in-mouth perceived body was also previously reported [62], confirming the release of wood extractives with such impact [58]. An interesting aspect of the results here obtained is the very short time needed to obtain perceivable variations of these descriptors. The first principal component (56.1 % of variability) accounted to variability mainly related to time and micro-oxygenation. The control wine resulted the poorest as regards aroma and taste properties. The effect of passive micro-oxygenation, without the concurrent activity of wood extractables and tannins was therefore detrimental for wine sensory profile, as expected [63]. Interestingly, the sensory impact of the low-pressure extraction process resulted durable and tolerated the impact of oxygenation, especially when oenological tannin was added [45].

**Fig. 9:**
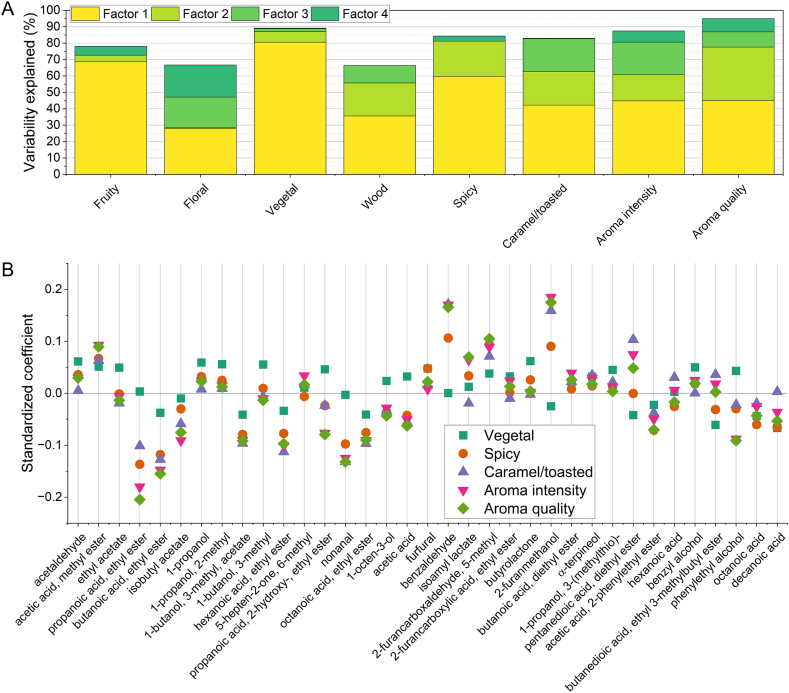
Results of the partial least squares regression (PLSR) analysis of scores of aroma descriptors as a function of levels of volatile compounds in wine. Plot of the cumulative percent variance of each Y (descriptors) explained by the model with 4 factors (A); plot of the standardized coefficients of each volatile compound in the models of descriptors with explained variance ≥80 % (B).

Finally, partial least squares regression (PLSR) analysis was carried out to relate the volatile profiles of wines with their aroma properties, including both wines after dynamic extraction and wines after passive micro-oxygenation. Fig. 9 reports the results of the multivariate regression analysis. The root mean predicted residual of sum of squares (PRESS) and the analysis of percent cumulative variance explained indicated that 4 factors could provide the best explanatory performance of the aroma descriptors as a function of volatile profile. The variability of some descriptors could be better explained by the volatile profile analysed (Fig. 9A). In fact, fruity and floral notes could be explained for less than 80 % of their variability. Unexpectedly, also the wood notes were poorly represented by the obtained model. This could probably be due to the underestimation of lactones in the volatile profile analysis, since literature reports a strong relationship between this class of compounds and the intensity of wood perception [54]. On the other hand, the variability of herbal, spicy, caramel/toasted perceptions, as well as aroma intensity and quality could be explained for more than 80 % on the basis of the data of volatile compounds.

The impact of individual compounds on the models can be evaluated as standardized coefficients (Fig. 9B). Coefficients are reported only for the descriptors whose variability can be explained for more than 80 % by the volatiles considered. A substantial concordance among coefficients of spicy, caramel/toasted, aroma intensity and aroma quality could be observed. All these descriptors showed negative coefficients for ethyl esters of short- and medium-chain fatty acids and for acetates, while positive coefficients were assigned to furan compounds, in particular 5-methyl-furfural and 2-furanmethanol. A relatively high coefficient for these descriptors was assigned to benzaldehyde, frequently associated to almond odor [56,64]. Diethyl-glutarate (pentanedioic acid, diethyl ester) showed also positive coefficients for these descriptors. It has been reported by Garde-Cerdán et al. [65] as a compound increasing during barrel aging. Authors hypothesized that glutaric acid could form during malolactic fermentation. As regards the herbal note, the main contributors to such perception were higher alcohols, showing positive coefficients.

## 4 Conclusions

The adoption of a low-pressure continuous dynamic extraction process to foster the interaction between Primitivo wine and oak chips allowed to improve color and to modulate the volatile profile and the sensory properties of wine, in few hours. When combined with passive micro-oxygenation through permeable plastic materials and oenological tannin, a relevant improvement in color stabilization could be obtained, without compromising the aroma profile. These tools can provide an integrated technological platform suitable to tune wine sensory properties and stability, when either traditional approaches (such as barrel aging) or other assisted extraction technologies are not applicable or preferred.

## Ethical statement

No human ethics committee or formal documentation process is available. Participants to sensory analysis gave informed consent via the statement "I am aware that my responses are confidential, and I agree to participate in this survey" where an affirmative reply was required to enter the survey. They were able to withdraw from the survey at any time without giving a reason. The products tested were safe for consumption.

## Data availability

The data associated with this study has not been deposited into a publicly available repository. Data will be made available on request.

## CRediT authorship contribution statement

**Vito Michele Paradiso:** Writing – original draft, Visualization, Supervision, Methodology, Conceptualization. **Gabriele Fioschi:** Writing – review & editing, Investigation, Data curation. **Massimo Tripaldi:** Supervision, Resources, Conceptualization. **Luigi Sanarica:** Writing – review & editing, Supervision, Resources, Conceptualization. **Chiara Pisarra:** Writing – review & editing, Investigation, Data curation. **Mirella Noviello:** Writing – review & editing, Investigation, Data curation. **Ilaria Prezioso:** Writing – review & editing, Investigation, Data curation. **Giuseppe Gambacorta:** Writing – review & editing, Resources, Methodology.

## Declaration of competing interest

The authors declare that they have no known competing financial interests or personal relationships that could have appeared to influence the work reported in this paper.

## Figures and Tables

**figs1:**