# Supplementary material for: Low-pressure continuous dynamic extraction from oak chips combined with passive micro-oxygenation to tune red wine properties
Source: Heliyon. 2024 Aug 10;10(16):e36100. doi: 10.1016/j.heliyon.2024.e36100 (PMC11367132; doi:10.1016/j.heliyon.2024.e36100)
Supplement: Multimedia component 1 [file mmc1.docx]

Supplementary table S1. Results of two-way ANOVA and post-hoc Tukey’s test for multiple comparisons for the chemical parmeters of the analysed wines.^a^

***Redox potential***

|  | **DF** | **Sum of Squares** | **Mean Square** | **F Value** | **P Value** |
| --- | --- | --- | --- | --- | --- |
| Extraction time | 5 | 3458.20345 | 691.64069 | 7.14491 | <0.0001 |
| Storage | 2 | 10181.20982 | 5090.60491 | 52.5879 | <0.0001 |
| Model | 7 | 16639.68169 | 2377.09738 | 24.55633 | <0.0001 |
| Error | 40 | 3872.0731 | 96.80183 |  |  |
| Corrected Total | 47 | 20511.75479 |  |  |  |

*Tukey’s test – Extraction time*

| **Hours** | **Mean** | **Groups** | | | |
| --- | --- | --- | --- | --- | --- |
| 7 | 164.9875 | A |  |  |  |
| 4 | 154.91111 | A | B |  |  |
| 5.5 | 148.53333 |  | B | C |  |
| 2.5 | 143.25556 |  | B | C |  |
| 1 | 140.62222 |  |  | C |  |
| 0 | 120.475 |  |  |  | D |

*Tukey’s test – Storage*

| **Treatment** | **Mean** | **Groups** | |
| --- | --- | --- | --- |
| 4 months + tannin | 161.44 | A |  |
| 4 months | 158.45625 | A |  |
| t0 | 125.34118 |  | B |

***Dissolved oxygen***

|  | **DF** | **Sum of Squares** | **Mean Square** | **F Value** | **P Value** |
| --- | --- | --- | --- | --- | --- |
| Extraction time | 5 | 9.11983 | 1.82397 | 6.28179 | 2.17397E-4 |
| Storage | 2 | 0.06849 | 0.03424 | 0.11794 | 0.88906 |
| Model | 7 | 9.1358 | 1.30511 | 4.49486 | 9.04894E-4 |
| Error | 40 | 11.61429 | 0.29036 |  |  |
| Corrected Total | 47 | 20.7501 |  |  |  |

*Tukey’s test – Extraction time*

| **Hours** | **Mean** | **Groups** | | |
| --- | --- | --- | --- | --- |
| 7 | 2.69875 | A |  |  |
| 4 | 2.26333 | A | B |  |
| 5.5 | 1.95556 | A | B | C |
| 2.5 | 1.64 |  | B | C |
| 0 | 1.5225 |  | B | C |
| 1 | 1.46667 |  |  | C |

*Tukey’s test – Storage*

| **Treatment** | **Mean** | **Groups** |
| --- | --- | --- |
| 4 months + tannin | 1.972 | A |
| 4 months | 1.95375 | A |
| t0 | 1.92765 | A |

***Color density***

|  | **DF** | **Sum of Squares** | **Mean Square** | **F Value** | **P Value** |
| --- | --- | --- | --- | --- | --- |
| Extraction time | 5 | 3.51304 | 0.70261 | 1.02671 | 0.41472 |
| Storage | 2 | 60.24141 | 30.1207 | 44.015 | <0.0001 |
| Model | 7 | 69.9192 | 9.98846 | 14.59601 | <0.0001 |
| Error | 41 | 28.05745 | 0.68433 |  |  |
| Corrected Total | 48 | 97.97665 |  |  |  |

*Tukey’s test – Extraction time*

| **Hours** | **Mean** | **Groups** | |
| --- | --- | --- | --- |
| 7 | 10.1095 | A |  |
| 5.5 | 9.43767 | A | B |
| 4 | 9.391 | A | B |
| 2.5 | 9.34011 | A | B |
| 1 | 9.14433 | A | B |
| 0 | 8.3906 |  | B |

*Tukey’s test – Storage*

| **Treatment** | **Mean** | **Groups** | **Groups** |
| --- | --- | --- | --- |
| 4 months + tannin | 11.10553 | A |  |
| 4 months | 8.71735 |  | B |
| t0 | 8.46288 |  | B |

***Color density (corrected)***

|  | **DF** | **Sum of Squares** | **Mean Square** | **F Value** | **P Value** |
| --- | --- | --- | --- | --- | --- |
| Extraction time | 5 | 1.40562 | 0.28112 | 1.29232 | 0.28594 |
| Storage | 2 | 51.25527 | 25.62763 | 117.80897 | <0.0001 |
| Model | 7 | 56.3836 | 8.0548 | 37.02752 | <0.0001 |
| Error | 41 | 8.91896 | 0.21754 |  |  |
| Corrected Total | 48 | 65.30256 |  |  |  |

*Tukey’s test – Extraction time*

| **Hours** | **Mean** | **Groups** | |
| --- | --- | --- | --- |
| 7 | 9.57488 | A |  |
| 5.5 | 9.29422 | A |  |
| 4 | 9.17389 | A |  |
| 1 | 9.02922 | A | B |
| 2.5 | 8.97233 | A | B |
| 0 | 8.3578 |  | B |

*Tukey’s test – Storage*

| **Treatment** | **Mean** | **Groups** | **Groups** |
| --- | --- | --- | --- |
| 4 months + tannin | 10.7074 | A |  |
| 4 months | 8.47447 |  | B |
| t0 | 8.34929 |  | B |

***Hue***

|  | **DF** | **Sum of Squares** | **Mean Square** | **F Value** | **P Value** |
| --- | --- | --- | --- | --- | --- |
| Extraction time | 5 | 0.0015 | 3.00923E-4 | 0.20626 | 0.95799 |
| Storage | 2 | 0.07286 | 0.03643 | 24.96979 | <0.0001 |
| Model | 7 | 0.07859 | 0.01123 | 7.69573 | <0.0001 |
| Error | 41 | 0.05982 | 0.00146 |  |  |
| Corrected Total | 48 | 0.13841 |  |  |  |

*Tukey’s test – Extraction time*

| **Hours** | **Mean** | **Groups** |
| --- | --- | --- |
| 4 | 1.0382 | A |
| 7 | 1.03281 | A |
| 1 | 1.03037 | A |
| 5.5 | 1.02951 | A |
| 2.5 | 1.02036 | A |
| 0 | 0.9995 | A |

*Tukey’s test – Storage*

| **Treatment** | **Mean** | **Groups** | **Groups** | **Groups** |
| --- | --- | --- | --- | --- |
| 4 months + tannin | 1.0763 | A |  |  |
| 4 months | 1.03227 |  | B |  |
| t0 | 0.9784 |  |  | C |

***Resistant pigments***

|  | **DF** | **Sum of Squares** | **Mean Square** | **F Value** | **P Value** |
| --- | --- | --- | --- | --- | --- |
| Extraction time | 5 | 0.4969 | 0.09938 | 3.57779 | 0.00891 |
| Storage | 2 | 6.2532 | 3.1266 | 112.56004 | <0.0001 |
| Model | 7 | 7.50271 | 1.07182 | 38.58623 | <0.0001 |
| Error | 41 | 1.13886 | 0.02778 |  |  |
| Corrected Total | 48 | 8.64158 |  |  |  |

*Tukey’s test – Extraction time*

| **Hours** | **Mean** | **Groups** | | |
| --- | --- | --- | --- | --- |
| 7 | 2.69263 | A |  |  |
| 4 | 2.55233 | A | B |  |
| 5.5 | 2.54433 | A | B |  |
| 2.5 | 2.43867 |  | B |  |
| 1 | 2.338 |  | B | C |
| 0 | 2.1304 |  |  | C |

*Tukey’s test – Storage*

| **Treatment** | **Mean** | **Groups** | **Groups** | **Groups** |
| --- | --- | --- | --- | --- |
| 4 months + tannin | 2.9762 | A |  |  |
| 4 months | 2.45576 |  | B |  |
| t0 | 2.04784 |  |  | C |

***Chemical age***

|  | **DF** | **Sum of Squares** | **Mean Square** | **F Value** | **P Value** |
| --- | --- | --- | --- | --- | --- |
| Extraction time | 5 | 0.0098 | 0.00196 | 1.33336 | 0.26946 |
| Storage | 2 | 0.10438 | 0.05219 | 35.48711 | <0.0001 |
| Model | 7 | 0.12153 | 0.01736 | 11.8049 | <0.0001 |
| Error | 41 | 0.0603 | 0.00147 |  |  |
| Corrected Total | 48 | 0.18182 |  |  |  |

*Tukey’s test – Extraction time*

| **Hours** | **Mean** | **Groups** |
| --- | --- | --- |
| 7 | 0.57097 | A |
| 4 | 0.5671 | A |
| 5.5 | 0.55939 | A |
| 2.5 | 0.55634 | A |
| 1 | 0.52691 | A |
| 0 | 0.51767 | A |

*Tukey’s test – Storage*

| **Treatment** | **Mean** | **Groups** | **Groups** |
| --- | --- | --- | --- |
| 4 months | 0.59186 | A |  |
| 4 months + tannin | 0.58054 | A |  |
| t0 | 0.4867 |  | B |

| ^a^, Means that do not share a letter are significantly different. |
| --- |
