# Supplementary material for: Low-pressure continuous dynamic extraction from oak chips combined with passive micro-oxygenation to tune red wine properties
Source: Heliyon. 2024 Aug 10;10(16):e36100. doi: 10.1016/j.heliyon.2024.e36100 (PMC11367132; doi:10.1016/j.heliyon.2024.e36100)
Supplement: Multimedia component 2 [file mmc2.docx]

| Supplementary table S2. Oxygen permeation in PET bottles filled with a model wine | | | |
| --- | --- | --- | --- |
| Dissolved oxygen (mg L^-1^) | | Oxygen intake (mg L^-1^ day^-1^) | Acetaldehyde (mg L^-1^) |
| At filling | After 14 days |  | After 14 days |
| 0.75±0.05 | 4.02±1.22 | 0.23±0.08 | N.D. |
