# Supplementary material for: Low-pressure continuous dynamic extraction from oak chips combined with passive micro-oxygenation to tune red wine properties
Source: Heliyon. 2024 Aug 10;10(16):e36100. doi: 10.1016/j.heliyon.2024.e36100 (PMC11367132; doi:10.1016/j.heliyon.2024.e36100)
Supplement: Multimedia component 3 [file mmc3.docx]

| Supplementary table S3. List of the volatile compounds analysed in the wines, with retention times.^a^ | |
| --- | --- |
|  | Retention time (min) |
| Aldehydes | |
| Acetaldehyde | 4.8 |
| Nonanal | 25.3 |
| Benzaldehyde | 31.0 |
| Benzeneacetaldehyde | 35.8 |
| Alcohols | |
| 1-Propanol | 10.5 |
| 1-Propanol, 2-Methyl | 12.3 |
| 1-Butanol, 3-Methyl | 17.0 |
| 1-Octen-3-Ol | 27.3 |
| Benzyl Alcohol | 44.4 |
| Phenylethyl Alcohol | 45.6 |
| Ethyl Esters | |
| Propanoic Acid, Ethyl Ester | 8.3 |
| Butanoic Acid, Ethyl Ester | 8.5 |
| Hexanoic Acid, Ethyl Ester | 18.3 |
| Propanoic Acid, 2-Hydroxy-, Ethyl Ester | 23.1 |
| Octanoic Acid, Ethyl Ester | 27.0 |
| Butanoic Acid, Diethyl Ester | 36.8 |
| Pentanedioic Acid, Diethyl Ester | 40.7 |
| Acetate Esters | |
| Methyl Acetate | 5.9 |
| Ethyl Acetate | 6.8 |
| Isobutyl Acetate | 9.8 |
| 1-Butanol, 3-Methyl, Acetate | 13.7 |
| Acetic Acid, 2-Phenilethyl Ester | 42.2 |
| Other Esters | |
| Isoamyl Lactate | 32.3 |
| Butanedioic Acid, Ethyl 3-Methylbutyl Ester | 45.0 |
| Terpene Compounds | |
| 5-Hepten-2-One, 6-Methyl | 22.9 |
| α-Terpineol | 37.6 |
|  | |
| Acetic Acid | 28.0 |
| Hexanoic Acid | 43.1 |
| Octanoic Acid | 50.4 |
| Decanoic Acid | 57.0 |
| Furan Compounds | |
| Furfural | 28.5 |
| 2-Furancarboxaldehyde, 5-Methyl | 33.0 |
| 2-Furancarboxylic Acid, Ethyl Ester | 34.5 |
| 2-Furanmethanol | 36.3 |
| Lactones | |
| Butyrolactone | 35.4 |
| Sulfur Compounds | |
| 1-Propanol, 3-(Methylthio)- | 38.5 |
